# Hybrid Nitric Oxide Donor and its Carrier for the Treatment of Peripheral Arterial Diseases

**DOI:** 10.1038/s41598-017-08441-9

**Published:** 2017-08-18

**Authors:** Duong Q. Le, Aneetta E. Kuriakose, Dat X. Nguyen, Kytai T. Nguyen, Suchismita Acharya

**Affiliations:** 1 Department of Bioengineering, University of Texas at Arlington, Arlington, TX 76010 USA; 2 Joint Biomedical Engineering Program, University of Texas Southwestern Medical Center, Dallas, TX 75390 USA; 3 North Texas Eye Research Institute, University of North Texas Health Science Center, Fort Worth, TX 76107 USA

## Abstract

Nitric oxide (NO) has been known to promote physiological angiogenesis to treat peripheral arterial diseases (PAD) by increasing the vascular endothelial growth factor (VEGF) level in endothelial cells (ECs) and preventing platelet adherence and leukocyte chemotaxis. However, the ongoing ischemic event during peripheral ischemia produces superoxide and diminishes the NO bioavailability by forming toxic peroxynitrite anion. Here we disclose an efficacious hybrid molecule 4-(5-Amino-1,2,3-oxadiazol-3-yl)-2,2,6,6-tetramethyl-1-piperidinol (SA-2) containing both antioxidant and NO donor functionalities that provide a therapeutic level of NO necessary to promote angiogenesis and to protect ECs against hydrogen peroxide-induced oxidative stress. Compound SA-2 scavenged reactive oxygen species, inhibited proliferation and migration of smooth muscle cells (SMCs) and promoted the tube formation from ECs. Copolymer poly(lactic-co-glycolic acid) (PLGA) nanoparticles loaded with SA-2 provided a sustained release of NO over days, improved aqueous stability in serum, protected ECs against oxidative stress, and enhanced angiogenesis under stress conditions as compared to that of the control in the *in vitro* matrigel tube formation assay. These results indicated the potential use of SA-2 nanoparticles as an alternative therapy to treat PAD.

## Introduction

Peripheral arterial diseases (PAD), the occlusive arterial disorder in the lower extremities of the body, is prevalent in elderly people. According to American Heart Association statistics, about 8.5 million Americans over 40 suffer from PAD^1^, and it manifests in ~10% of individuals greater than 65 years old and ~20% of individuals over 80 years of age. Of the high PAD mortality rates, 13,854 American deaths in 2010 were recorded^1^. Ischemia related to PAD occlusions has high rates of amputations and mortalities worldwide. Common treatments such as bypass grafts, endovascular and percutaneous interventions are feasible methods in restoring sufficient perfusion to maintain normal vessel functions, yet they often cause frequent late thrombosis and restenosis in arteries^2, 3^. These facts indicate the importance in the development of an alternative therapy to treat PAD.

Recently, therapeutic angiogenesis, the sprouting of new blood vessels from pre-existing vasculatures^4^, has proven to be a potential strategy to mitigate PAD patients’ symptoms as it promotes vessel formation and lowers blood pressure while supplying oxygen-rich blood and nutrients to tissues in deficits^5^. This treatment route requires the administration of exogenous pro-angiogenic factors to trigger EC proliferation and migration and to remodel the extracellular matrix (ECM) for tubule formation and expansion^6^. Another strategy is targeting the nitric oxide-cyclicguanosine monophosphate (NO-cGMP) pathway^7–9^. Nitric Oxide (NO) acts as an important signaling molecule regulating vascular inflammation^10, 11^, platelet function^11^, angiogenesis^12^, and protection from ischemia reperfusion injury^13, 14^; however, it is impaired in PAD^15^. Currently, many research studies have shown that although the supply of NO is important to ECs, it is crucial to maintain NO concentration at the physiological level. Excessive NO supply expands the NO gradient between extracellular and endogenous levels that consequently leads to ROS elevation, EC dysfunction^16^ and poor endothelial progenitor cells’ (EPCs) viability^17^. These observations suggest that using a NO donor that can provide a physiological concentration of NO might be effective for the treatment and prevention of PAD.

Besides regulating NO levels, reactive oxygen species (ROS) also play an important role in PAD pathology^18^. The ROS, including superoxide (O_2_^−^), peroxynitrite (ONOO^−^), hydroxyl group (OH^.^) and hydrogen peroxide (H_2_O_2_), are regulated by body antioxidants such as superoxide dismutase, glutathione, glutathione peroxidase, catalase and so on^19^. In peripheral ischemia, a shortage of oxygen puts cells under oxidative stress, which in turn elevates ROS levels at the injured site. In addition, EC dysfunction at the injured site causes inflammation that recruits immune cells and produces more ROS. Excess superoxide also diminishes the NO bioavailability by forming toxic ONOO^−^. When the ROS level exceeds the antioxidants produced in the body, more EC dysfunction and smooth muscle cell (SMC) recruitment might occur^20^. Consequently, the accumulation of EC dysfunction and SMC migration leads to atherosclerosis and/or chronic inflammatory conditions^21^. Thus, the incorporation of antioxidant functionality to a NO donor is expected to reduce damage in ECs from oxidative stress^22, 23^.

**Figure 1:**
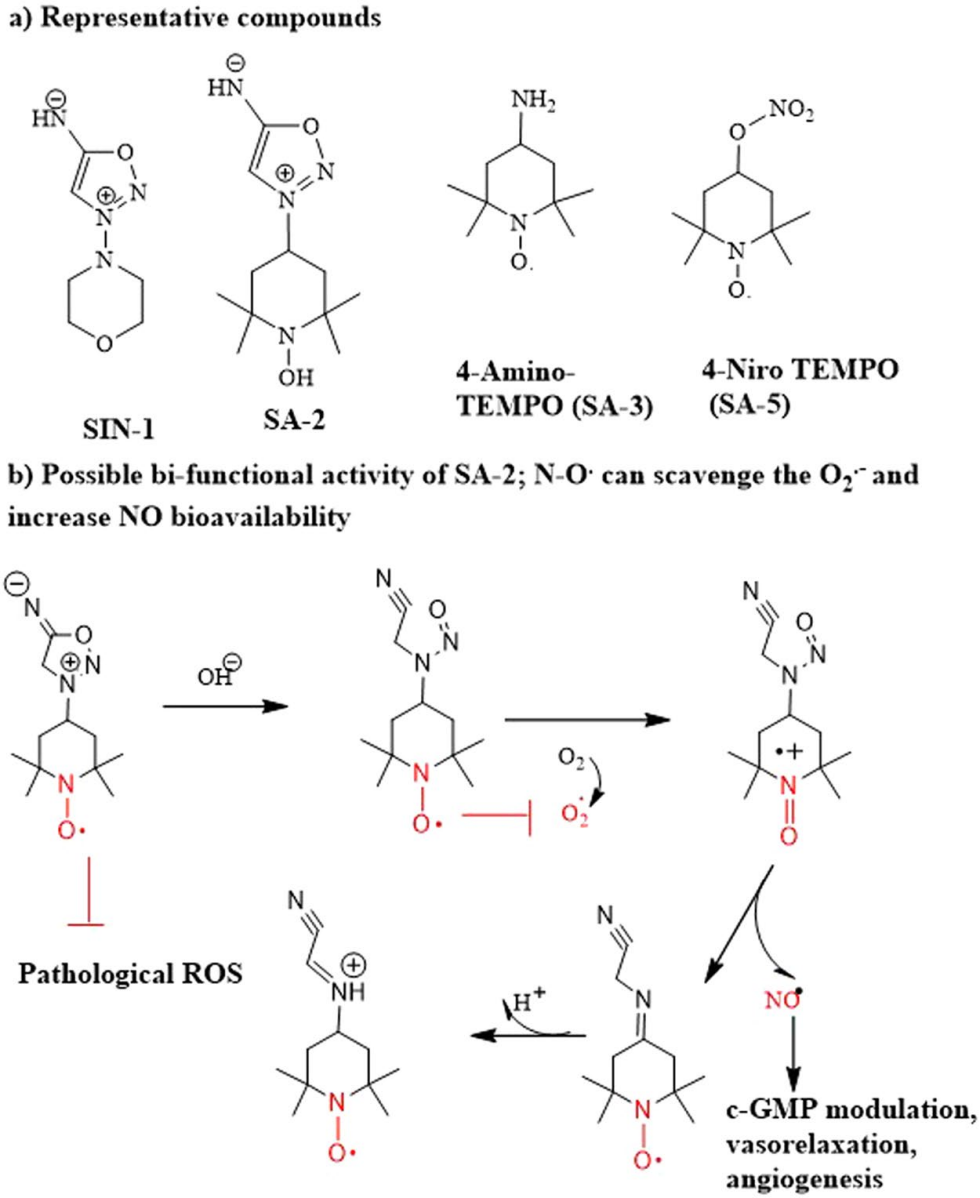
(**a**) SIN-1 (reference NO donor), SA-2 (Hybrid compound), SA-3 (reference antioxidant), SA-5 (reference hybrid NO donor-antioxidant). (**b**) Possible mechanism of synergistic activity of compound SA-2.

In the present study, we investigated the approach of combining “spontaneous” or pH-responsive NO donor^24^ and superoxide dismutase (SOD) mimetic (nitroxide) functional groups (SA-2, Fig. 1a) to both maintain a therapeutic level of NO and scavenge superoxide^25^, respectively. We have also studied the effects of standard pH-responsive NO donor (3-(4-Morpholino)-sydnonimine hydrochloride, SIN-1, Fig. 1a), SOD mimetic nitroxide (4-Amino TEMPO, SA-3, Fig. 1a) or mitochondrial enzymes-dependent No donor hybrid compound^26^ (4-nitro TEMPOL, SA-5, Fig. 1a) to compare their effects and validate the advantages of our hybrid molecule SA-2 on inhibiting SMC migration and proliferation as well as preventing EC dysfunction. A sustainable delivery system consisting of SA-2, SA-2-loaded PLGA nanoparticles (SA-2 NPs), was also synthesized, characterized and confirmed for a sustained release of SA-2 to further provide the improved EC survival and enhanced angiogenesis under stress conditions.

## Results

### Effects of SA-2 on the properties of ECs under stress conditions

**Figure 2:**
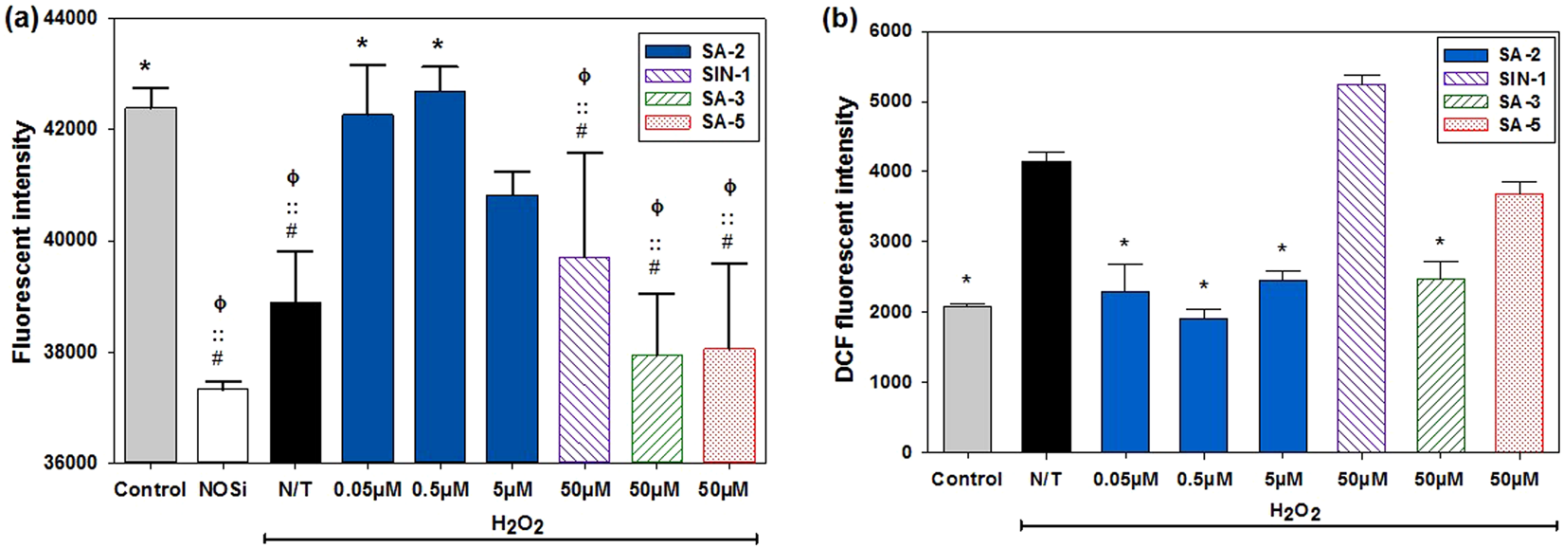
Effects of SA-2 on Nitric Oxide Synthase (NOS) activities (**a**) and scavenging of ROS (**b**) in HUVECs. The cells were seeded and after being confluent in tissue culture plates, they were co-treated with H_2_O_2_ and either SA-2 at different concentrations, a reference NO donor SIN-1 (50 µM), a reference antioxidant SA-3 (50 µM) or a reference hybrid compound SA-5 (50 µM). Control samples were cells without exposure to H_2_O_2_ and any reagent, whereas N/T samples were cells exposed to H_2_O_2_ only. NOS knock-down samples (NOSi) were cells exposed to a competitive NOS inhibitor L-NNA at 50 µM. After 24-hour-treatment, total NOS activity (Fig. 2a) from treated cells was quantified using OxiSelect™ Intracellular Nitric Oxide (NO) Assay Kit where NOS activity correlates to fluorescent intensity. Similarly, ROS levels (Fig. 2b) were quantified with DCFDA assays. Results were analyzed on SigmaPlot with ANOVA and post hoc Pairwise Multiple Comparisons using Holm-Sidak method. Data were shown as mean ± standard deviation. Stars (*), phi (ϕ), double-colon (::) and hashtag (#) indicate significant difference (P < 0.01; n = 4) with respect to N/T, control, SA-2 at 0.05 µM and SA-2 at 0.5 µM, respectively.

In Human Umbilical Vascular Endothelial Cells (HUVECs) under H_2_O_2_ induced oxidative stress condition, the hybrid compound SA-2 decreased the production of ROS and maintained a physiologically relevant level of eNOS (Fig. 2). In this experiment, SIN-1, a pure NO donor, at a concentration of 50 μM although produced more than 90 times of NO than that of SA-2 in cell culture media (data not shown), it failed to recover intracellular eNOS levels for ECs (Fig. 2a). Additionally, the hybrid compound SA-2 at concentrations ranging from 0.05 μM–5.0 μM effectively scavenged the excess ROS produced and maintained the redox status similar to the control (Fig. 2b), while the reference antioxidant SA-3 needed 10- to 100-fold more concentration to achieve such effects. We also noticed that the reported NO donor antioxidant hybrid SA-5 is less effective in decreasing the ROS level.

**Figure 3:**
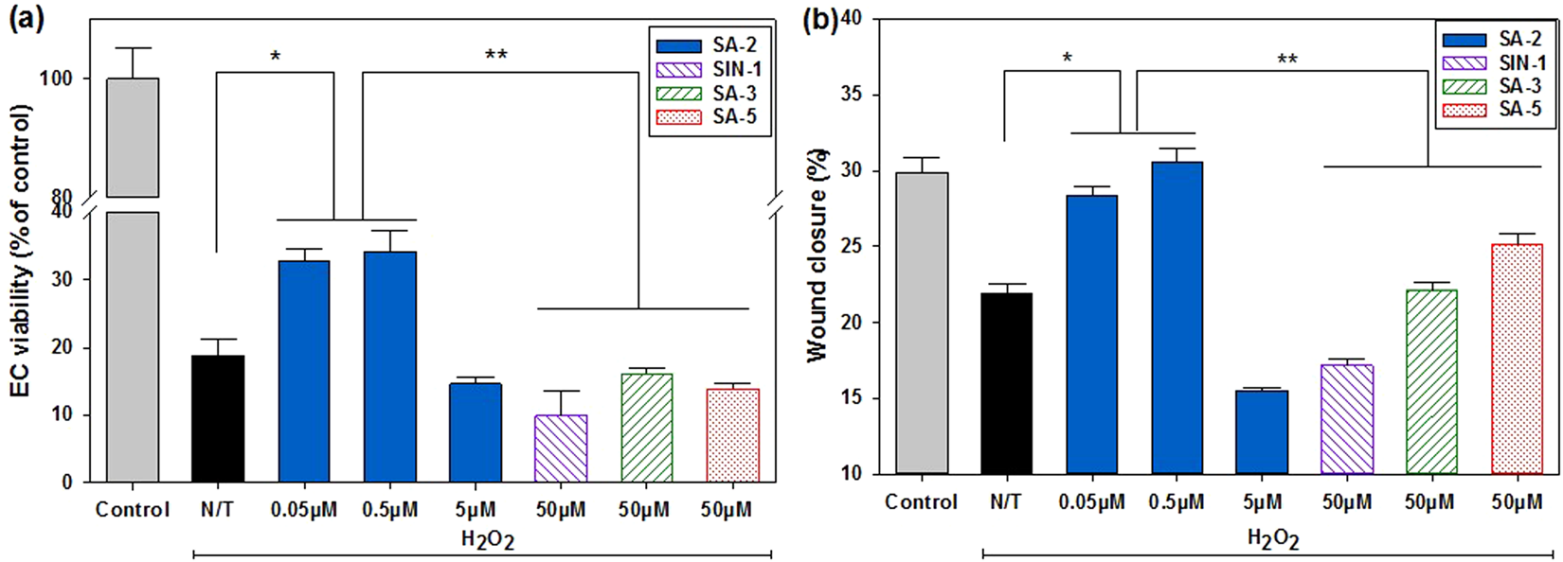
Effects of SA-2 on EC viability (**a**) and migration (**b**) under oxidative stress conditions. Cells were seeded and allowed to attach on tissue culture plates. For the cell migration study, micropipette tips were used to make scratch lines on wells, and images were taken for measuring distances of gaps. In all studies, cells were treated with H_2_O2, and SA-2 at different concentrations. A reference NO donor SIN-1 (50 µM), a reference antioxidant SA-3 (50 µM), or a reference hybrid compound SA-5 (50 µM) was added to cell samples, incubated for 24 hours, assessed for cell viability and migration, and used for comparison. Controls were cells not exposed to H_2_O_2_ or any treatment reagent. N/T samples were cells exposed to H_2_O_2_ without any test compound. Cell viability was quantified with MTS assays, while cell migration was imaged and analyzed for final distances of gaps via ImageJ. Results were then processed for statistical analysis using ANOVA followed by post-hoc comparisons (SigmaPlot). Results are presented as mean values ± SEM. Stars indicate significant differences (P < 0.05; n = 5) with respect to N/T (*) and reference drugs (**).

Dysfunction of ECs resulting from oxidative stress is a major contributor of impaired vascular endothelial growth factor (VEGF) production and new vessel formation. With the ability to maintain NO and ROS at the physiological level, compound SA-2 with EC_50_ of 0.354 μM (Supplementary Fig. S1) protected HUVECs from H_2_O_2_-induced oxidative stress, demonstrating the increase in cell viability, whereas SIN-1, which produced the elevated NO and ROS levels *in vitro*, turned out to induce more cell death (Fig. 3a). Additionally, as shown in Fig. 3b, there is good correlation between SA-2 and EC functions as shown in wound closure assessments (detail images shown in Supplementary Fig. S2) in the migration study and the cytoprotection study of HUVECs (Fig. 3a). As observed, cells treated with a low dose of SA-2 (0.05 μM–0.5 μM) migrated similarly to the control group (Fig. 3b), while SIN-1 showed the lowest cell migration. SA-3 and SA-5 showed an intermediate effect, but at a higher concentration (50 μM).

### Effects of SA-2 on the formation of new blood vessels

**Figure 4:**
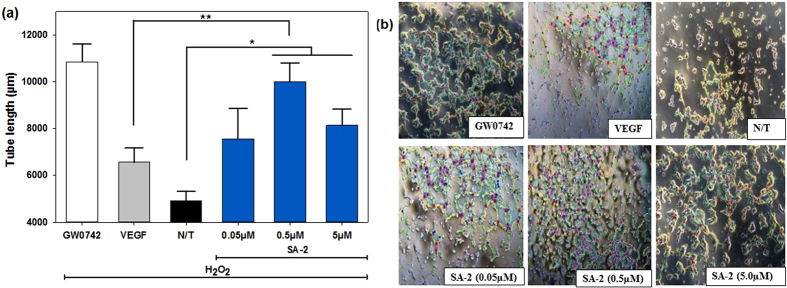
SA-2 promoted angiogenesis in ECs under an oxidative stress condition: (**a**) tube length and (**b**) representing images for angiogenesis analysis. Cells were seeded on Cultrex gel on tissue culture plates and stressed with H_2_O_2_. SA-2 at different concentrations, G0742 (1 µM), or VEGF (25 ng/mL) was added to each well. N/T samples were cells exposed to H_2_O_2_ only. After 8 hours, at least 10 images were randomly captured on a phase contrast microscope for each well (n = 4 wells/sample). Images were analyzed and quantified for length of microtubes formed using ImageJ with Angiogenesis Analyzer tools. Results were then processed on SigmaPlot for statistical analysis using ANOVA followed by post hoc comparisons. Data are shown as mean values ± SEM. Stars indicate significant differences (p < 0.05) with respect to N/T (*) and VEGF (**).

Compound SA-2 controls the ROS production, maintains NO level and protects ECs under stress conditions. Here we further performed the tube formation assay to evaluate the ability of SA-2 to promote angiogenesis under the same H_2_O_2_ induced oxidative stress condition. As expected, we found that, SA-2 successfully promoted new blood vessel formation (Fig. 4) and was more efficacious than VEGF (25 ng/mL). Compound SA-2 was also found to be equally potent to GW0742 (1 µM), a peroxisome proliferator activating receptor (PPAR) β/δ agonist^27^, in inducing angiogenesis.

### Effects of SA-2 on inhibiting the proliferation and migration of SMCs

**Figure 5:**
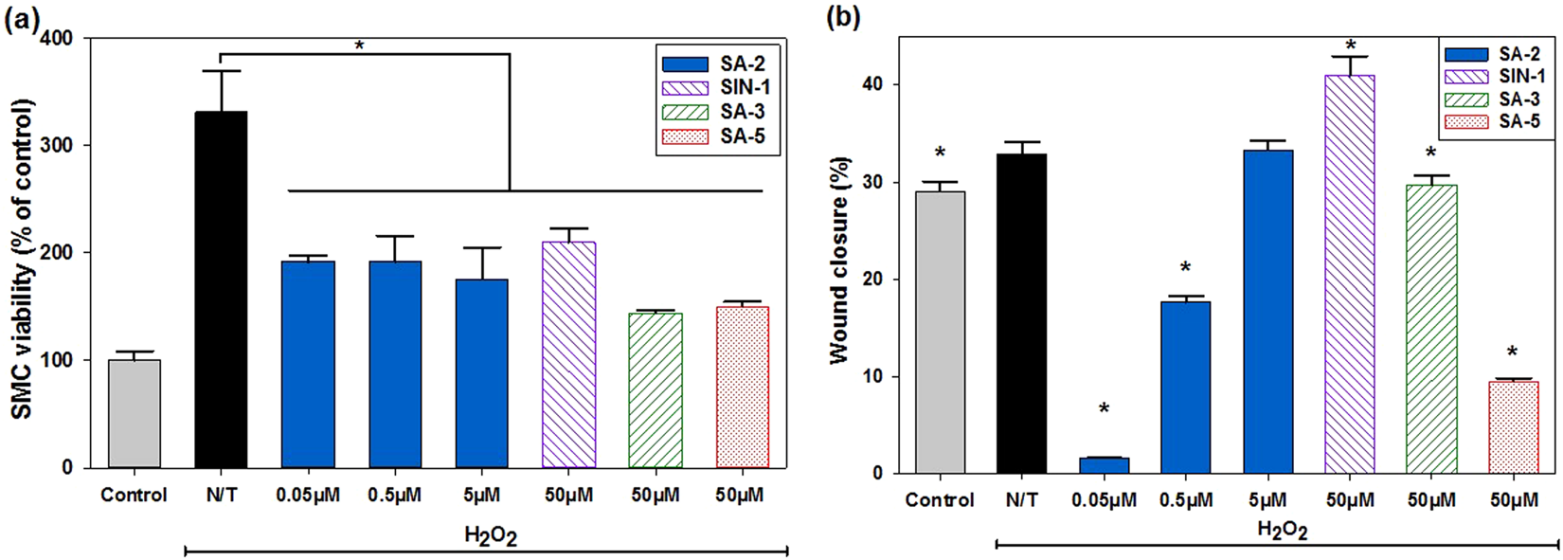
Effects of SA-2 on the SMC viability (**a**) and migration (**b**) under oxidative stress conditions. Cells were seeded and allowed to attach on tissue culture plates. For the migration study, micropipette tips were used to make scratch lines on wells, followed by washing with PBS and imaging for initial gap distances. Compound SA-2 at different concentrations, SIN-1 (50 µM), SA-3 (50 µM), or SA-5 (50 µM) was added to cell samples, stressed with H_**2**_O_**2**_ and incubated for 24 hours. Controls were cells not exposed to H_2_O_2_ or any treatment reagent. N/T samples were cells exposed to H_2_O_2_ without any test compound. Cell viability was quantified with MTS assays while cell migration was imaged and analyzed for final gap distances via ImageJ. Results were then processed on SigmaPlot for statistical analysis using ANOVA followed by post hoc comparisons. Results are presented as mean values ± SEM. Stars indicate significant differences (P < 0.05; n = 4) with respect to N/T.

In addition to increasing viability and promoting new blood vessel formation from HUVECs, hybrid compound SA-2 (0.05 µM–0.5 µM) also demonstrated substantial reduction in proliferation and migration of Human Aortic Smooth Muscle Cells (HASMCs) under stress conditions (Fig. 5). SMCs are three times more likely to grow under stress conditions compared to the normal state (Fig. 5a). The addition of NO donor, ROS scavenger, or hybrid compounds inhibited the proliferation of SMCs at the rate of about 50%. In addition, a significant reduction in migration of SMCs was observed when cells were treated with SA-2 with concentrations of 0.05 μM and 0.5 μM (Fig. 5b).

Cumulatively, we observed a good correlation between the NO production, scavenging of ROS, and the ability to protect the HUVEC death as well as promotion for wound healing under oxidative stress conditions by hybrid compound SA-2. The NO donor SIN-1 is previously reported to prevent EC damage due to ischemia and reperfusion^28^ and attenuated SMC activation by Interferon-γ induced VCAM-1 inhibition^29^ at millimolar concentrations. However, SIN-1 neither protected ECs nor promoted migration and wound closure when tested at a concentration of 50 μM in our study, whereas the hybrid compound SA-2 was highly effective in a nanomolar concentration. Additionally, in the *in vitro* matrigel tube formation assay in HUVECs, compound SA-2 (0.5 μM) demonstrated better potency to VEGF and was comparable to a PPAR δ/γ agonist GW0742 (1 μM). Under the same experimental condition, a pure antioxidant SA-3 or hybrid compound SA-5 were unable to protect the HUVECs from H_2_O_2_-induced cell death. In SMCs, compound SA-2 (0.05–0.5 μM) demonstrated superior activities in inhibiting the cell proliferation and migration under oxidative stress conditions. We have also observed that even though SIN-1 and SA-3 at concentrations of 50 μM were able to inhibit SMC proliferation, both of them did not inhibit cell migration shown as % of wound closure in Fig. 5b.

**Figure 6:**
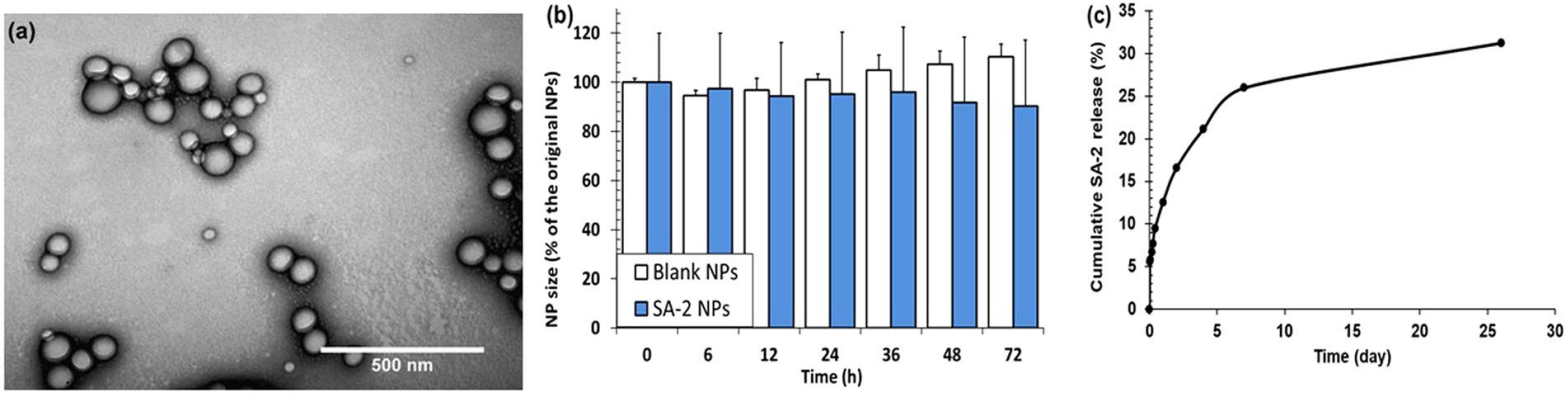
Characterization of SA-2 NPs. (**a**) Transmission electron microscopic (TEM) image, (**b**) stability in serum at pH_7.4_ over 3 days and (**c**) accumulative SA-2 release from SA-2 NPs at pH_7.4_ measured as absorbance measurements. Data (**b**,**c**) shown as mean ± SD, n = 4.

Despite of these exciting findings, compound SA-2 undergoes fast hydrolysis (t_1/2_ less than one day at pH_7.4_), which is common for most of the 1,3,5-oxadiazoles containing “spontaneous” NO donating compounds^25^. To improve the therapeutic bioavailability and the aqueous chemical stability of SA-2, it was encapsulated into FDA approved PLGA nanocarriers using a standard emulsion method similar to our previous studies^30–33^. PLGA NPs have been shown to protect degradation and inactivity of various therapeutic reagents, including SA-2, and to extend the therapeutic efficacy as observed by other investigators and our group. As seen from the TEM image (Fig. 6a), SA-2 NPs are homogenously dispersed, ranging from 90 to 150 nm. The TEM size is in harmony with an average diameter of 173 ± 35 nm measured with a dynamic light scattering (DLS) technique. Zeta potential of these NPs was −38 ± 0.14 mV. A stability study in serum demonstrated that these NPs were stable and did not aggregate over 3 days (Fig. 6b). SA-2 was loaded in PLGA carriers at loading efficiency of 56% containing 70 µg of SA-2 per 1 mg of NPs. In addition, a biphasic sustained release profile of SA-2 was obtained via quantification of SA-2 released from NPs over the time (Fig. 6c). The biphasic release consisted of a burst release of 16% of SA-2 within 2 days and a sustained release up to 35% of SA-2 over a month. The improvement in stability and sustained SA-2 release of drug carriers is a significant achievement to aid drug delivery to PAD patients via either intravenous (IV) or intramuscular injections.

**Figure 7:**
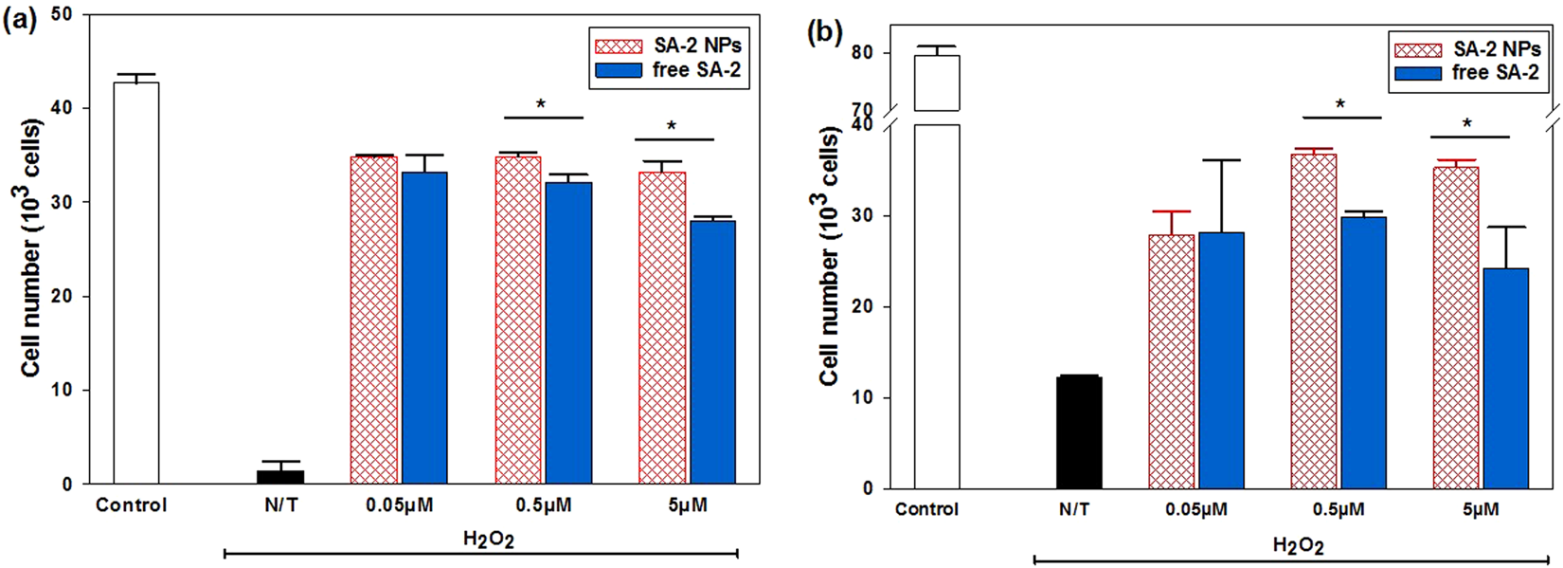
Effects of SA-2 NPs on HUVECs compared to that of free SA-2 under H_2_O_2_ induced oxidative stress conditions after (**a)** 1 day and (**b**) 4 days of treatment. Cells were seeded and allowed to attach on tissue culture plates. Cell samples were added to either SA-2 or SA-2 NPs at different concentrations, stressed with H_2_O_2_ and incubated for 1 day or 4 days. Controls were cells not exposed to stress or any treatment reagent. N/T samples were cells exposed to H_2_O_2_ without any test compound. Cell numbers for studied groups were analyzed on SigmaPlot with ANOVA followed by post hoc comparisons. Results are presented as mean values ± SD. Stars (*) indicates significant differences (P < 0.05; n = 4) with respect to free drug at the same concentration.

Under oxidative stress conditions, SA-2 NPs at a concentration range of 0.05 μM-5.0 μM exhibited superior effects over free SA-2 by increasing ECs viability after 1 day of treatment and the effect was continued to 4 days of post treatment (Fig. 7). It is reasonable to argue that, free SA-2 was quickly hydrolyzed in less than a day so only a small percentage of SA-2 was responsible for EC protection, whereas the NPs slowly release SA-2 to continuously induce the effect on ECs. As expected SA-2 NPs demonstrated more effective EC protection (EC_50_ at 0.1 µM, Supplementary Fig. S3) than free SA-2 (EC_50_ at 0.354 µM, Supplementary Fig. S3) under oxidative stress condition.

### Effects of SA-2 NPs on angiogenesis in HUVECs

**Figure 8:**
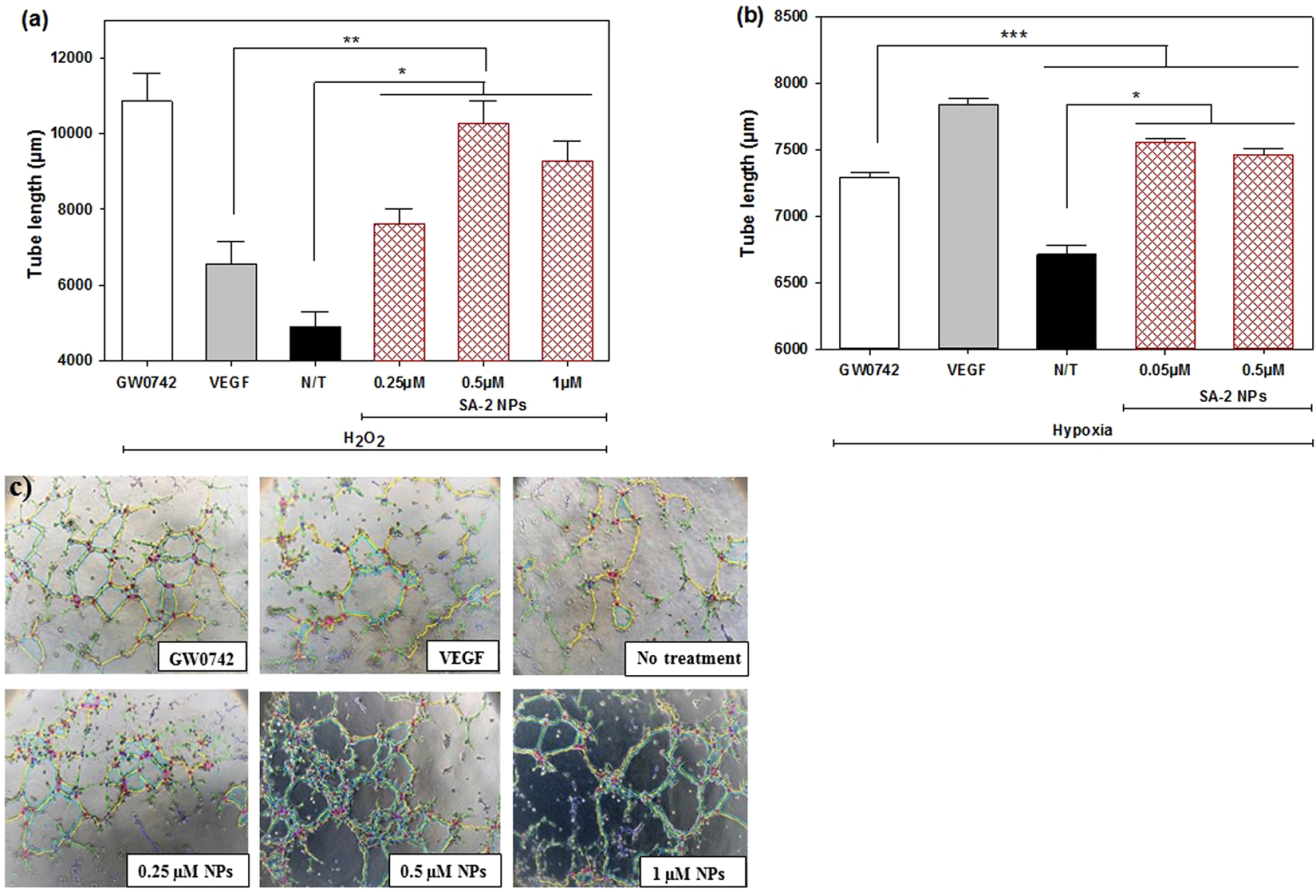
Effects of SA-2 NPs on angiogenesis of ECs under oxidative and hypoxic stress conditions. Cells were seeded on Cultrex gel on tissue culture plates and stressed with either (**a**) H_2_O_2_ or (**b**) hypoxia. SA-2 NPs at different concentrations, GW0742 (1 µM) or VEGF (25 ng/ml), were added to each well. N/T samples were cells exposed to (**a**) H_2_O_2_ only or (**b**) hypoxia without any treatment. After 8 hours of treatment, at least 10 images were randomly captured on a phase contrast microscope for each well. (**c**) Angiogenesis under H_2_O_2_ induced oxidative stress conditions analyzed by ImageJ. Images were analyzed and quantified for length of microtubes formed using ImageJ with Angiogenesis Analyzer tools. Numeric data were then analyzed on SigmaPlot with ANOVA followed by post hoc comparisons. Results are shown as mean values ± SEM. Stars indicate significant differences (P < 0.01; n = 3) with respect to N/T (*), VEGF (**) and GW0742 (***).

As expected, SA-2 NPs were effective in promoting *in vitro* angiogenesis similar to that of the parent drug SA-2 (Fig. 8a vs. Fig. 4a). In the *in vitro* matrigel tube formation study, SA-2 NPs significantly promoted angiogenesis in terms of tube length under both oxidative (Fig. 8a) and hypoxic (Fig. 8b) stress conditions.

## Discussion

Compounds activating the NO–cGMP pathway have been shown to be vasorelaxing, angiogenic and protective against EC dysfunction. The additional benefits on the inhibition of SMC proliferation and migration have also been reported. Comprehensive studies on soluble guanylate cyclase sGC-cGMP pathway have revealed that not only NO bioavailability is important but its concentration is also physiologically crucial. At low concentrations (nano to low micromolar), NO exhibits cytoprotection such as vasodilatation of SMCs and proliferation of ECs^34, 35^. However, higher concentrations of NO lead to reverse effects, including peroxynitrite radical formation, protein nitrosylation, and apoptosis. Hence, it is necessary to balance between superoxide and NO bioavailability at the injured arteries in order to maintain the vascular homeostasis.

Reported literature on many NO donors indicated effective concentrations in ranges from 50 to 100 µM; therefore, we chose 50 µM as a concentration for reference drugs (SIN-1, SA-3 and SA-5) in our studies. In the study by Nguyen *et al*.^16^ a dose- and time-dependent study was performed on HUVECs, demonstrating that NO donor SIN-1 at 50 µM exhibited maximal effects on HUVEC functions. Results were quantified in terms of L-arginase activity, which contributes crucially as a cofactor with endothelial NO synthase (eNOS) under the same low serum conditions as used in our study. They also reported that although SIN-1 produced NO extracellularly, it diminished L-arginase activities and decreased endogenous NO produced by ECs^16^. To support this further, our results also demonstrated that, cells under oxidative stress conditions reduced NOS activities (thus decreased NO production) similar to that of cells treated with the NOS inhibitor (L-NNA). Additionally, SIN-1 and other reference drugs were not able to recover NOS levels to that of the control group. This might be due to mitochondria dysfunction as mentioned in literature^17, 36^. In contrast, SA-2 at 0.05 µM and 0.5 µM maintain eNOS levels equal to that of controls. As a result, EC viability, migration and angiogenesis for cells treated with SA-2 were increased while ones treated with reference drugs were not. Similar to our findings, a low dose of an NO donor DCBPY (cis-[Ru(H-dcbpy^−^)_2_(Cl)(NO)]) to HUVECs provided NO at the same level as the control groups, and therefore induced relaxation of aortic rings and improved EC functions^37^. This result is in support of our results where SA-2 at 0.5 µM provided a physiological NO production, improved EC viability, and facilitated angiogenesis under stress conditions.

During PAD, ischemic events induce oxidative stress resulting in EC dysfunction via decreased activity of antioxidant enzymes in mitochondria. Such events of mitochondrial dysfunction and production of superoxide diminish the NO bioavailability by reacting with NO and forming toxic ONOO^−^ that further damages DNA, lipids and proteins^17^. ROS and ONOO^−^ specifically dysfunctionalize ECs and increase the endo-exogenous NO imbalance. Consequently, we believe that there exists a loop where excessive NO produces more stress of ROS on ECs. Therefore, besides balancing NO levels, it is also crucial to regulate ROS levels under ischemic conditions. A study by Nguyen *et al*.^16^ was in correlation with our study that SIN-1 elevated the ROS level in mouse aortic ECs under the stress conditions. A similar study was reported where exposure of bovine aortic endothelial cells (BAECs) to SIN-1 at 100 µM-250 µM damaged mitochondria and induced apoptosis^36^, and this effect was reversed with the help of an antioxidant diphenyl diselenide (PhSe)_2_ at 1 µM concentration. Other common antioxidants are TEMPOL derivatives that possess variable protection against nitration and thiol oxidation that are both induced by peroxynitrite^38^. In addition, the saturated protection (90%) and half protection (50%) were observed at 100 µM and 50 µM of TEMPO, respectively^38^. It was therefore reasonable for us to choose the concentration for antioxidant drugs SA-3 and SA-5 as 50 µM for our studies to see a maximal effect.

The idea of combining NO donor and ROS scavenging has been reported elsewhere in literature, either with physical combination or chemical (hybrid compound) combination. The former idea reported by Park *et al*.^39^ showed that the combination of SIN-1 (100 µM) and TEMPO (100 µM) reduced pulmonary vascular resistance more than the single effect of SIN-1 (100 µM). In the same study, a similar trend was also reported for a decrease in both platelet recruitment^39^ and thrombotic von Willebrand factor (an important indication in PAD) secretion. Similar to our study, Das *et al*.^40^ investigated the effect of a cyclic hybrid compound DMPO containing NO radical on regeneration of BAECs. Cells when stressed with ONOO^•−^ and H_2_O_2_ and post-treated with DMPO showed dose- and time-dependent recovery after treatment with 50 µM of DMPO for 24 hours or 100 µM DMPO for 12 hours. In comparison, our SA-2 and SA-2 NPs are more potent and efficacious at concentrations of 0.025 µM to 0.5 µM, which are significantly lower doses. For SA-2 NPs, the protective efficacy was maintained, possibly due to the sustained NO release over a long period.

In addition to cell viability and apoptosis, the promotion of angiogenesis by antioxidants is reported by Dong *et al*.^41^ where endothelial progenitor cells pretreated with 10 µM of commercial antioxidant FeTMPyP recovered angiogenesis at about 50%. Here we found that the hybrid compound SA-2 increased the length of tube formation from ECs nearly two-fold as compared to the non-treatment group. Compared to positive controls, our hybrid compounds demonstrated comparable angiogenic effects to that of VEGF and a PPAR δ agonist GW0742.

The utilization of nanoparticles to deliver active molecules to treat PAD has been reported elsewhere in literature, for instance by Kwon *et al*.^42^ where poly(oxalic acid) NPs were utilized to deliver antioxidant vanillyl alcohol, and the results on HUVEC viability were dose-dependent with an increase in the NP concentrations (25 µg/mL to 100 µg/mL). This result is analogous to our finding that, the SA-2 NPs were more efficacious than the parent compound SA-2 in maintaining the EC viability and promoting migration at 24 hours. These results suggest that angiogenic effects *in vivo* would be improved by our SA-2 NPs, which will be investigated in the future.

In summary, we have demonstrated that, the hybrid molecule SA-2 containing redox catalytic antioxidant and NO donor functionality showed better *in vitro* efficacy than a pure NO donor SIN-1, pure antioxidant SA-3 or another literature hybrid compound SA-5. Compound SA-2 promoted angiogenesis and protected ECs against oxidative stress with EC_50_ of 0.354 μM. The hybrid compound SA-2 scavenged the ROS, inhibited SMC proliferation and migration, and promoted tube formation from ECs. Similar to other NO donors such as GSNO, diazonium diolates, and sydnonimines, compound SA-2 would utilize pH-dependent hydrolysis to provide spontaneously large amounts of NO at pH_7.4_ in a short period of time, leading to a short duration of therapeutic activity. To circumvent this problem and improve the therapeutic efficacy, we have successfully synthesized and characterized the SA-2-loaded PLGA NPs. These NPs were stable in serum and provided sustained release of SA-2 over days. These SA-2 NPs were also found to be efficacious in protecting HUVECs against H_2_O_2_ induced oxidative stress with EC_50_ of 0.1 μM and more potent in promoting angiogenesis as compared to that of VEGF. Evaluation of SA-2 NPs in a mouse model of hind limb ischemia is in progress.

## Methods

### Chemicals and reagents

Synthesis of compound SA-2 was described previously^25^. Compounds SIN-1 and SA-5 were prepared with >95% purity^43, 44^. Compounds SA-3, GW0742 and Carboxy-H_2_DCFDA were purchased from TCI (Portland, OR), Tocris Bioscience (Bristol, UK) and Molecular Probes (Paisley, UK) respectively. Chloroform and hydrogen peroxide (3%) were bought from Sigma-Aldrich (St. Louis, MO). PLGA with copolymer ratio 50:50 and inherent viscosity of 0.4 dL/g was received from Lakeshore Biomaterials (Birmingham, AL). MTS (CellTiter 96®Aqueous One Solution Cell Proliferation Assay) assay kits, Cultrex Basement Membrane and Extract OxiSelect™ Intracellular Nitric Oxide (NO) Fluorometric Assay Kit were purchased from Promega (Madison, WI), Trevigen (Gaithersburg, MD) and Cell Biolabs (San Diego, CA) respectively.

### Fabrication of SA-2 NPs

SA-2 loaded PLGA NPs were prepared using the standard single emulsion technique developed in our laboratory^30–33, 45–49^. In brief, 10 mg of SA-2 was dissolved in 3 ml of chloroform containing 100 mg of PLGA to form an oil phase. This solution was then added dropwise into 20 ml of 5% PVA solution (water phase) and emulsified at 30 W for 5 minutes to form the SA-2 loaded nanoparticles. The final emulsion was stirred overnight to allow solvent evaporation. The nanoparticles were washed and collected by ultracentrifugation and lyophilized before use.

### Characterization of SA-2 NPs

Size of SA-2 NPs were characterized by dynamic light scattering (Brookhaven Instruments, ZetaPALS) and TEM images (Hitachi, H-9500). The stability of SA-2 NPs in media with serum were quantified by ZetaPALS in terms of the size change after incubating of these NPs in the solvent over predetermined time points. The drug loading efficiency and drug release of SA-2 NPs were quantified by absorbance measurement of SA-2 contents. In brief, SA-2 standard solutions was subjected to wavelength absorbance scanning where wavelength of 230 nm gave the peak reading. Absorbance at 230 nm of SA-2 at different concentrations were used for plotting a SA-2 standard curve with a linear regression equation. Using this equation, amount of unloaded SA-2 in supernatant was measured and the loading efficiency was calculated as 56% using the following equation:

1 $$Loading\,efficiency( \% )\,\,\frac{Total\,amount\,of\,drug\,used-Unloaded\,amount\,of\,drug}{Total\,amount\,of\,drug\,used}\times 100 \% $$

For the drug release study, SA-2 NPs were suspended in the phosphate buffer saline (PBS) solution at 1 mg/ml concentration, placed in a dialysis bag with MWCO 5000 Da, and incubated at 37 °C over a time range. At each time point, a fixed volume of dialysate solution was pooled and replaced with the same volume of fresh PBS. The absorbance value of each sampling solution was read and the amount of released SA-2 was quantified. Consequently, a cumulative release profile of SA-2 was plotted. Study was repeated in triplicate and data plot was mean values ± standard error.

The aqueous chemical stability of SA-2 (t_1/2_) was determined using Liquid Chromatography-Mass Spectroscopy on Shimadzu LC/MS-2020, while circulation stability of NPs was quantified in terms of size change by DLS as mentioned earlier.

### Doses and groups of study

For all studies, the no treatment (N/T) group was cells exposed to H_2_O_2_ only, while cells grown in complete media without exposure to anything served as a control group. The test compound, SA-2, was studied at concentrations of 0.05 µM, 0.5 µM and 5 µM. Compounds SIN-1, SA-3 and SA-5 were studied at a concentration of 50 µM according to their effective concentrations as mentioned in the discussion section. For therapeutic study of SA-2 NPs, the concentration of NPs used was based on the released SA-2 amount equivalent to the amount of free SA-2. Accordingly, SA-2 NPs were used at SA-2 concentrations of 0.05 µM, 0.5 µM and 5 µM for all studies except the angiogenesis study (where 0.05 µM, 0.25 µM, 0.5 µM and 1 µM were used).

### Media and cell lines

HUVECs and HASMCs were purchased from American Type Culture Collection (ATCC, Manassas, VA), while culture media (Vasculife Basal Medium) and supplemental kits (Vasculife VEGF Lifefactors for HUVEC, Vasculife SMC Lifefactors for HASMC) were purchased from Lifeline Cell Technology (Frederick, MD). HUVECs and HASMCs cultured in complete media with 2% and 5% supplemented serum, respectively, were used as controls for our experimental studies. For the experiments, all the treatment groups (SA-2, SIN-1, SA-3, SA-5 and SA-2 NPs) under stress conditions were cultured in low serum media (0.2% and 0.5% supplemented serum for HUVECs and HASMCs, respectively) at 37 °C.

### Cellular stress conditions

Cell behavior was investigated under stress conditions with exposure to either H_2_O_2_ or hypoxia.

#### Oxidative stress

Pilot studies with different concentrations of H_2_O_2_ (0–400 µM) were performed with ECs over various time ranges (4–24 hours), and the cell viability was assessed by MTS assays. Results showed that exposure to 400 µM H_2_O_2_ in 24 hours significantly reduced EC viability (Supplementary Fig. S4) and was chosen as an oxidative stress condition for ECs experiments associated with cell viability. In the angiogenesis and migration studies, the concentration of H_2_O_2_ was reduced to 200 µM to avoid biased results due to the cell death (Supplementary Fig. S4).

#### Hypoxic stress

Cells were subjected to 1% O_2_ and 5% CO_2_ at 37 °C to mimic chronic hypoxia in PAD per literature protocol^50^, whereas the cells exposed to 21% O_2_ and 5% CO_2_ served as a control (normoxic condition).

### Assessment of eNOS levels in EC

Cells were seeded and allowed to attach on tissue culture well plates at a density of 20,000 cells/cm^2^. After incubation, cells were co-treated with H_2_O_2_ and either SA-2 at different concentrations, SIN-1 or SA-5 for 24 hours. NOS reference group is cells treated with the NOS inhibitor L-NNA (N5-[imino(nitroamino)methyl]-L-ornithine) at 50 µM and no H_2_O_2_. After 24 hours of incubation, cells were washed several times with Dulbecco’s Phosphate-Buffered Saline, and the eNOS level in cell sample was quantified with Intracellular Nitric Oxide Fluorometric Assay Kit following manufacturer’s instruction. In brief, the NO probe (provided with the kit) diffuses into cells and is deacetylated by cellular esterases to a non-fluorescent intermediate, then it is rapidly oxidized by intracellular nitric oxide to a triazolo-fluorescein analog and emits high fluorescence. The fluorescence intensity is proportional to the NO levels within the cell cytosol.

### Determination of ROS Activity

Cells were co-treated with H_2_O_2_ and compounds (SA-2 at different concentrations, SA-3 or SA-5). After 24 hours of treatment, cytosolic ROS levels were measured using Carboxy-H_2_DCFDA following the manufacturer’s protocol on a UV–vis spectrometer (Infinite M200 plate reader, Tecan, Durham, NC) at a wavelength of 485/530 nm (Excitation/Emission). Fluorescence intensities of DCF is proportionated to the cytosolic ROS.

### Cell Viability Studies

Cells (HUVECs or HASMCs) were co-treated with H_2_O_2_ and either the hybrid compound SA-2 or reference compounds (SIN-1, SA-3, or SA-5). Due to fast hydrolysis of spontaneous NO donor such as SA-2 and SIN-1, all groups were refreshed with treatment reagents every 12 hours. After 24 hours of treatment, cell viability was quantified using MTS assays following the manufacturer’s instructions.

### Cell Migration Studies

To understand the effects of SA-2 on the migration of HUVECs and HASMCs, an *in vitro* scratching assays were performed using these cells. 20,000 cells/well were seeded into 48-well tissue culture plates and incubated for 24 hours. Using a 1000 µl pipette tip, a 0.5 mm gap was made in each well, and images were captured using a phase contrast microscope. Cells were then co-treated with H_2_O_2_ and the test compounds as described earlier. All groups were refreshed with treatment reagents every 12 hours. After 24 hours of treatment, cells were stained with crystal violet and imaged again. The average distance or width of the gap before and after the treatment was determined using ImageJ software. The percentage of wound closure was quantified as below^51, 52^:

2 $${\rm{Wound}}\,\text{closure}\,( \% )=\frac{{\rm{Distance}}\,{\rm{before}}\,{\rm{migration}}-{\rm{Distance}}\,{\rm{after}}\,{\rm{migration}}}{{\rm{Distance}}\,{\rm{before}}\,{\rm{migration}}}\times 100$$

### Effects of SA-2 NPs on EC responses

HUVECs were exposed to SA-2 NPs, SA-2 drugs and co-treated with H_2_O_2_. After 1 and 4 days, cell viability was quantified using MTS assays and converted to a cell number based on the same linear fit curve of different cell numbers. EC viability was presented as number of cells that grew.

### *In-vitro* Angiogenesis Studies

Cultrex gel (liquid form) was coated on 24-well plates and allowed to gel for 30 minutes at 37 °C. HUVECs suspended in basal media (Vasculife) were seeded on gel coated well plates at a seeding density of 25,000 cells/cm^2^. The cells were co-treated with SA-2 and 200 µM H_2_O_2_. Cells treated with either GW0742 (1 µM) or VEGF (25 ng/ml) served as positive controls, whereas cells exposed to H_2_O_2_ at 200 µM without any treatment served as negative controls (N/T). After 8 hours of treatment, the tube formation was imaged randomly (at least 10 areas for each group) using a phase contrast microscope. As the gel starts to disintegrate at 16 hours, the angiogenesis study could not continue over an extended period of treatment.

For SA-2 NPs, cells were co-treated with 0.25, 0.5 and 1 µM and 200 µM of H_2_O_2_. A similar study was performed on NPs at 0.05 and 0.5 µM under hypoxic stress conditions. In this study, positive controls were cells treated with either GW0742 (1 µM) or VEGF (25 ng/ml) in hypoxic stress conditions while N/T was cells under hypoxic conditions without any treatment. The images taken were analyzed using angiogenesis analysis tools in ImageJ software, and the length of the tubes (µm) was measured to determine the potential angiogenesis.

### Statistical analysis

All the experiments were performed with n = 3–6. Data were expressed as mean ± SEM. The statistical analysis was assessed using ANOVA followed by post hoc Pairwise Multiple Comparisons using Holm-Sidak method on SigmaPlot version 13.0. A significant difference was considered where *P* values appeared ≤0.05.

### Data availability

All data generated or analyzed during this study are included in this published article (and its Supplementary Information files). The datasets generated during and/or analyzed during the current study are also available from the corresponding authors on reasonable request.

## Electronic supplementary material

Supplementary Figures
