# Supplementary material for: Hybrid Nitric Oxide Donor and its Carrier for the Treatment of Peripheral Arterial Diseases
Source: Sci Rep. 2017 Aug 18;7:8692. doi: 10.1038/s41598-017-08441-9 (PMC5562917; doi:10.1038/s41598-017-08441-9)
Supplement: Supplementary file 1 — Supplementary Figures [file 41598_2017_8441_MOESM1_ESM.pdf]

Hybrid Nitric Oxide Donor and its Carrier for the Treatment of Peripheral Arterial Diseases

Duong Q Le[a,b,1], Aneetta E Kuriakose[a,b,1], Dat X Nguyen[a,b], Kytai T Nguyen*[a,b] and Suchismita Acharya*[c]

[a]*Department of Bioengineering, University of Texas at Arlington, Arlington, TX 76010, USA*

[b]*Joint Biomedical Engineering Program, University of Texas Southwestern Medical Center, Dallas, TX 75390, USA*

[c]*North Texas Eye Research Institute, University of North Texas Health Science Center, Fort Worth, TX 76107, USA*

[1]Authors contributed equally; *corresponding authors

Supplementary Data

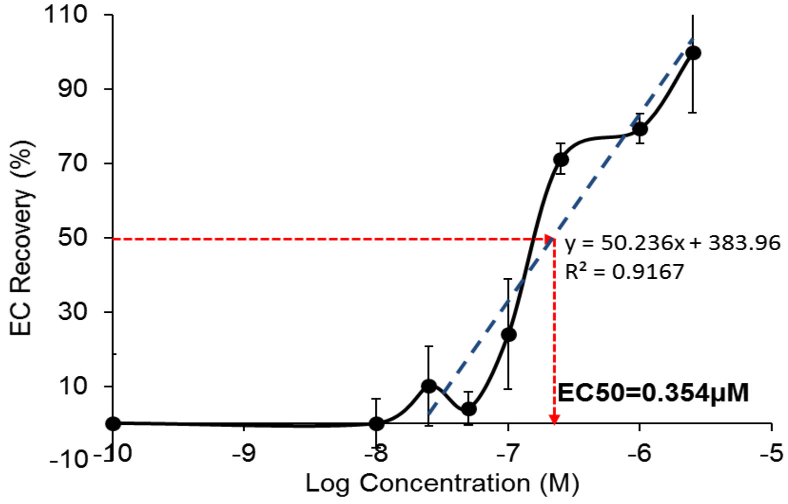

Figure S1: Dose response study of SA-2 on EC viability under oxidative stress. After seeding and being confluent in tissue culture plates, cells were treated with H_2O_2 at 400 µM and **SA-2** at eight different concentrations. Cells were refreshed with **SA-2**, H_2O_2 and corresponding media every 12 hours and incubated for a total of 24 hours. Cell viability was quantified with MTS assays. Data were shown as mean ± standard error against log10 of concentration (in Moles per Liter). Red arrows indicate 50% recovery and log concentration calculated. Linear fit was determined from a start concentration (at 0.025 µM) of minimal recovery (plateau at 0%) to maximal recovery (100% at 2.5 µM).

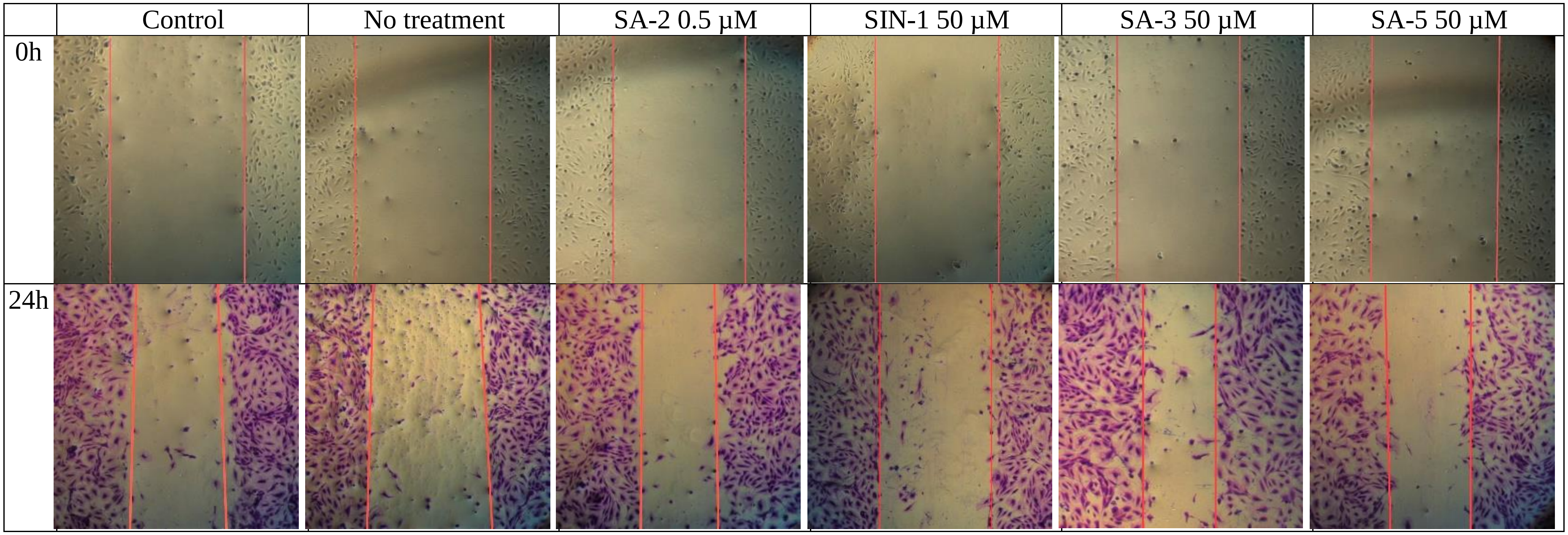

Figure S2: Representing migration images of ECs under stress conditions. Cells were seeded and allowed to attach on tissue culture plates. Next, micropipette tips were used to make scratch lines on plate surfaces, followed by washing with PBS to remove scratched cells. Then cell images were taken for initial gap distances (upper row, denoted as 0h). Cells were then treated with 200 µM H_2O_2 and followed by the addition of either **SA-2** at different concentrations, a reference NO donor SIN-1 (50 µM), a reference antioxidant SA-3 (50 µM) or a reference hybrid compound SA-5 (50 µM). Controls were cells not exposed to H_2O_2 or any reagent. No treatment group was cells exposed to H_2O_2 without any test compounds. Cells were refreshed with new media, test compounds and H_2O_2 in every 12 hours. Total incubation time was 24 hours. After incubation, cells were stained with crystal violet and imaged on a phase contrast microscope for the final distance of gaps (lower row, denoted as 24h). Red lines indicate borders of scratch. Images were quantified for gap distances at random spaces (at least 50 random measurements per each sample) on ImageJ.

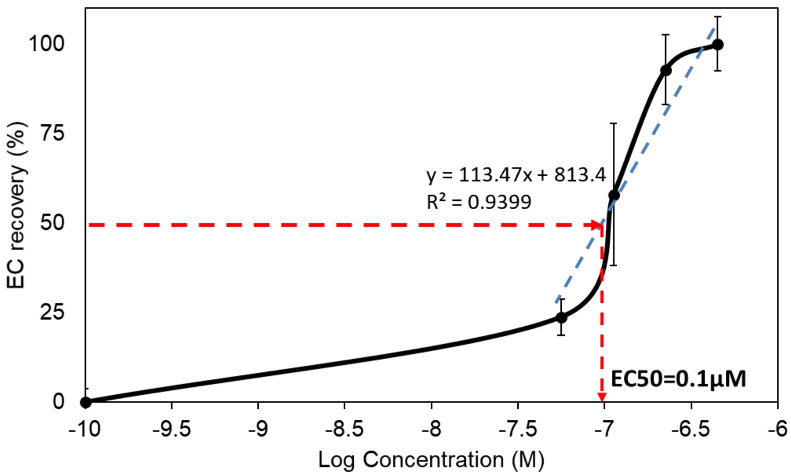

Figure S3: Dose response study of SA-2 NPs on EC viability under oxidative stress. After seeding and being confluent in tissue culture plates, cells were treated with H_2O_2 at 400 µM and SA-2 NPs at different concentrations. The concentration of NPs used was based on the released **SA-2** amount (24 hours for this study) equivalent to the amount of free **SA-2**. After 24 hours of incubation, cell viability was quantified with MTS assays. Data were shown as mean ± standard error against log10 of concentration (in Moles per Liter). Red arrows indicate 50% recovery and log concentration calculated. Linear fit was determined from the lowest concentration (0.08 µM) to the highest concentration (at 0.63 µM).

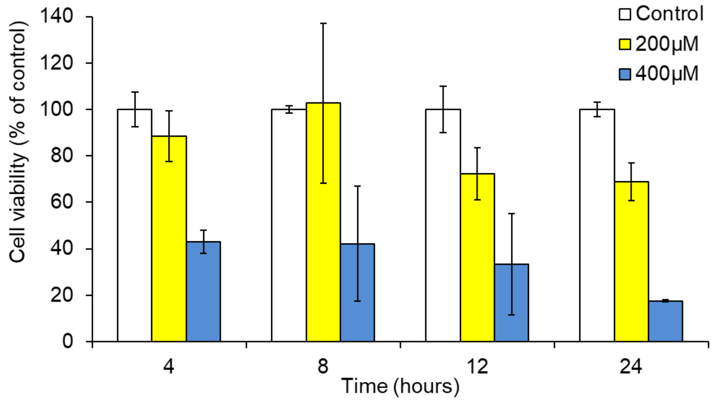

Figure S4: Effects of oxidative stress conditions on EC viability. Cells was seeded and allowed to attach on tissue cell culture plates. Next, cells were treated with different concentrations of H_2O_2. Cells in complete media without any stress served as a control. At predetermined time points, cell viability for each sample was quantified with MTS assays. Data presented as mean ± SD.
